# New Biomarker Combination Related to Oxidative Stress and Inflammation in Primary Open-Angle Glaucoma

**DOI:** 10.3390/life13071455

**Published:** 2023-06-27

**Authors:** Azza Dammak, Juan Sanchez Naves, Fernando Huete-Toral, Gonzalo Carracedo

**Affiliations:** 1 Ocupharm Group Research, Faculty of Optic and Optometry, Universidad Complutense de Madrid, 28037 Madrid, Spain; 2 Institute of Ophthalmology Palma de Mallorca, 07012 Palma de Mallorca, Spain; 3 Faculty of Optic and Optometry, Department Optometry and Vision, C/Arcos del Jalon 118, 28032 Madrid, Spain

**Keywords:** glaucoma, aqueous humor, oxidative stress, inflammation, biomarker

## Abstract

Glaucoma is a multifactorial neurodegenerative disease and the second leading cause of blindness. Detection of clinically relevant biomarkers would aid better diagnoses and monitoring during treatment. In glaucoma, the protein composition of aqueous humor (AH) is relevant for the discovery of biomarkers. This study analyzes AH protein concentrations of putative biomarkers in patients with primary open-angle glaucoma (POAG) compared to a control group. Biomarkers were selected from known oxidative-stress and inflammatory pathways. Osteopontin (OPN), matrix metalloproteinase 9 (MMP-9), tumor necrosis factor-alpha (TNF-alpha), transforming growth factor-beta (TGF-beta), and interleukin-10 (IL-10) were measured using the ELISA technique. Thirty-two patients were recruited to the study, including sixteen control and sixteen glaucoma patients. The glaucoma group consisted of patients diagnosed with glaucoma. In both groups, the aqueous humor sample was obtained during cataract surgery. A significant increase in OPN, MMP-9, TNF-alpha, and IL-10 was observed in the POAG aqueous humor, compared to the control group (*p* < 0.05). Of note, the AH of POAG patients contained 5.6 ± 1.2-fold more OPN compared to that of control patients. Different expression profiles of oxidative stress-related and inflammatory biomarkers were observed between patients with POAG and controls. This confirms the reported involvement of inflammatory and oxidative stress pathways in POAG pathophysiology. In the future, several, targeted AH proteins may be used to generate a potential biomarker expression profile of this disease, aiding diagnoses and disease progression monitoring. This approach highlights the importance of biomarkers in the future. Biomarkers provide a way to measure disease progression and response to treatment. In the future, biomarkers will play a more critical role in the toolkit of ophthalmology healthcare professionals as the field moves towards personalized medicine and precision healthcare.

## 1. Introduction

Glaucoma is one of the leading causes of vision loss globally [1]. It is an optic neuropathy identified by irreversible damage to the optic nerve head and death of retinal ganglion cells [2]. In developed countries, glaucoma is considered the second cause of irreversible blindness. In the United States and Europe, primary open-angle glaucoma (POAG), where the drainage channels within the eye become clogged, leading to increased intraocular pressure (IOP), is present in 75% of patients, making it the most common type of glaucoma in these populations [3].

The primary risk factor for POAG is elevated intraocular pressure (IOP) [4], due to trabecular meshwork (TM) dysfunction. TM is the main pathway for aqueous humor (AH) drainage [5]. The deposition of extracellular matrix proteins in the TM causes resistance to AH outflow and increases IOP in glaucoma. The AH leaves the eye via the TM and uveal-scleral pathways. Therefore, the drainage of the AH is important in glaucoma. The reason behind vision loss in POAG is the degeneration of retinal ganglion cells and the optic nerve. Perhaps, the root cause of this degeneration may be related to problems with the drainage of the AH in the anterior segment, increasing the IOP.

AH is composed primarily of water and trace amounts of vitamins, sugars, proteins, solutes, electrolytes, cytokines, and growth factors [6]. The AH is essential in facilitating the nutrient supply and removing metabolic wastes from the eye’s anterior chamber [6].

Mitochondrial dysfunction can lead to the generation of reactive oxygen species (ROS), causing oxidative stress. Apoptosis, tissue remodeling, transcription regulation, oxidative stress, and inflammation constitute the principal molecular mechanisms and pathways involved in the pathogenesis of glaucoma [7].

The inflammatory process results in the release of inflammatory cytokines and chemokines, which have been detected in the AH of POAG patients [8]. It has been demonstrated that elevated levels of the inflammatory cytokine tumor necrosis factor-alpha (TNF-α) can induce retinal ganglion cell (RGC) apoptosis in patients with glaucoma [9]. In addition, high levels of TNF-α in the aqueous humor have been associated with POAG, and its potential as a biomarker for glaucoma diagnoses or progression has been suggested [9,10,11]. Pro-inflammatory cytokine IL-10 concentration has been found to be higher in the AH of patients with primary open-angle glaucoma [8]. Also, changes in MMP-9 activity in the AH and tear samples of patients with POAG and early forms of primary angle-closure glaucoma (PACG) and POAG eyes compared to controls have been reported [12,13].

Osteopontin (OPN) is a member of the matricellular protein family and a vital regulator of the extracellular matrix (ECM) [14]. It was found to be increased in POAG and primary angle-closure glaucoma (PACG) compared to controls [15]. However, contradictory findings on the levels of OPN have been reported in glaucoma. OPN levels were significantly reduced in the AH of POAG patients compared to normal eyes [14]; however, OPN has been reported to be increased in the AH of a DBA2/J glaucoma mouse model relating to optic nerve damage. Recent studies have found an increase in oxidative stress in the retina and optic nerve head, contributing to the pathogenesis of glaucoma [16]. Furthermore, studying this biomarker requires investigations in the context of the oxidative stress pathway. Another biomarker studied in glaucoma is TGF-beta. TGF-beta plays a role in the deposition of extracellular material in space surrounding cells in various tissues, leading to increased resistance to aqueous humor outflow [17].

The selected biomarkers are essential constituents of cell membranes and organelles and are involved in biological processes and cell signaling. Detecting potential inflammatory and oxidative stress-related biomarkers in aqueous humor samples could provide essential information about the pathophysiology of glaucoma. This study evaluates the concentration of several relevant biomarker combinations in the aqueous humor of patients with glaucoma compared with patients not having glaucoma and considered as controls.

All glaucoma groups took IOP-lowering medications as preoperative preparation. IOP was evaluated, and the correlation between each biomarker related to glaucoma and IOP was studied. Moreover, in some studies, the levels of osteopontin have been evaluated by gender. This is because there may be differences in the expression and regulation of this protein between males and females, which can have implications for disease risk.

Osteopontin was studied, and the difference in the gender in both groups was evaluated.

## 2. Materials and Methods

### 2.1. Participants and Methods

This study was conducted at the Ocupharm research group laboratory (Faculty of Optics and Optometry, University Complutense of Madrid, Madrid, Spain) in collaboration with the Institute of Ophthalmology Palma de Mallorca (Palma de Mallorca, Spain). This study adhered to the Declaration of Helsinki in conducting experiments involving human subjects and was approved by the Clinical Research Ethics Committee of the University Complutense of Madrid (CEIC Clinico San Carlos, Madrid, Spain). Informed consent was obtained from each patient.

Aqueous humor samples were collected from patients (32 subjects) undergoing cataract surgery at the Institute of Ophthalmology Palma de Mallorca. The patients were divided into two groups, those diagnosed with POAG (study group) and patients who did not have glaucoma (control group) undergoing cataract surgery. The control group consisted of 16 patients undergoing cataract surgery. The other 16 patients presented cataracts with glaucoma. Both groups were matched for age and sex, discarding those patients whose clinical history reflects other traumatic cataracts, undergoing radiation therapy for cancer, or having a viral infection or ocular surgery other than cataract surgery procedure in the last 6 months before surgery and inclusion in this study. Patients with secondary glaucoma due to trauma and steroids-induced pseudoexfoliation were also excluded.

The inclusion criteria of the study group were over 40 years of age, being a candidate for cataract surgery, and diagnosis of POAG, including patients with glaucoma treatments. The control group included patients over 40 years of age, without concomitant ophthalmological pathology (other than cataract) and who underwent routine cataract surgeries without a history of other eye diseases or IOP exceeding 21 mmHg. Patients with a history of intraocular surgeries or local infections were excluded. Medical history and ophthalmologic examinations (visual acuity, slit lamp, and fundus examinations) were included for patients in both groups. IOP was evaluated before and after the surgery, and then aqueous humor was taken intraoperatively. IOP-lowering medications were given to all patients with glaucoma as preoperative prophylaxis. IOP was measured immediately before the operation.

Aqueous humor samples (between 100–200 µL) were collected in all patients with paracentesis, in an aseptic fashion, avoiding contact with the iris or limbal blood vessels. The aqueous humor was collected during the first step of cataract surgery. Following administration of an anesthetic drop (2% lidocaine), a 1 mm corneal microincision was made while visualizing the eye though a surgical microscope. A 30 gauge Rycroft cannula attached to a tuberculin syringe was used to aspirate the sample. After the cataract surgery, samples were immediately stored at −20 °C until their analysis.

### 2.2. Biomarker Measurement

AH samples were analyzed to measure the concentration of the following proposed biomarkers: osteopontin (OPN), matrix metalloproteinase 9 (MMP9), tumor necrosis factor-alpha (TNF-α), transforming growth factor beta (TGF-βeta) and interleukin-10 (IL-10). OPN, MMP-9, TNFA, TGF-beta 1, and IL-10 were analyzed using separate human ELISA kits according to the manufacturer’s protocol. ELISA kits used for the experiments were Human Osteopontin ELISA Kit Sigma-Aldrich, Human MMP-9 ELISA Kit Sigma-Aldrich, Human TNF-alpha ELISA Kit Sigma-Aldrich, Human TGF-beta ELISA Kit Sigma-Aldrich, and Human Il-10 ELISA Kit Sigma-Aldrich, St. Louis, MO, USA.

The volume of aqueous humor was diluted to analyze the five analytes using a small volume of sample with ELISA kits, and according to the provided collected volume (between 100 and 200 µL), the dilution factor was calculated to determine the final concentration. Each AH sample was diluted to reach the exact volume for ELISA technique. The number of patients for each biomarker corresponding to AH samples were different. For OPN and MMP-9, 16 AH samples were analyzed; for TGF-beta, 13 AH samples were analyzed and for TNF-alpha and IL-10, 8 AH samples were analyzed.

The experiments were realized in duplicate for each aqueous humor sample for the five biomarkers. The small volume of aqueous humor collected from patients was analyzed using the five ELISA kits. The samples were diluted to run all the experiments, with adequate provided buffer solution from ELISA kit, and the dilution factor was calculated to determine the real concentration for each biomarker.

For the correlation, some AH samples with very low concentration of the selected biomarker in both groups were excluded to analyze the correlation between each biomarker and IOP. The IOP average in this section is different between the 5 biomarkers.

Biomarker concentrations were determined by interpolating the measured absorbance intensities emitted from each sample against their standard curves. Gen 5 TM Manager software V 3.0 was used to read the absorbance using a BioTek Power Wave XS2 device (Bio Tek Instruments, Highland Park, Box 998 Winooski, VT, USA).

### 2.3. Statistical Analysis of the Data

To analyze the difference in the concentration of each biomarker between the control and patient groups, statistical differences between treatments were calculated using unpaired *t*-test. The data are presented as the mean ± SD of the results of the experiments. Plotting and fitting were carried out with GraphPad Prism version 9.3.1 for Windows (GraphPad Software, San Diego, CA, USA). The unpaired *t*-test was performed, and the significance level was set at *p* < 0.05. The correlation between two parameters (IOP and biomarker concentration) was determined with Pearson correlation. A *p*-value < 0.05 was considered statistically significant.

## 3. Results

### 3.1. Population Distribution and Clinical Characteristics

The mean age and sex of the study subjects were equivalent between control and glaucoma groups. The baseline preoperative intraocular pressure (IOP) and the postoperative IOP (Table 1; *p* = 0.3189) did not differ between the control and study groups.

### 3.2. Biomarker Quantification in the Aqueous Humor of the Study Population

**Figure 1:**
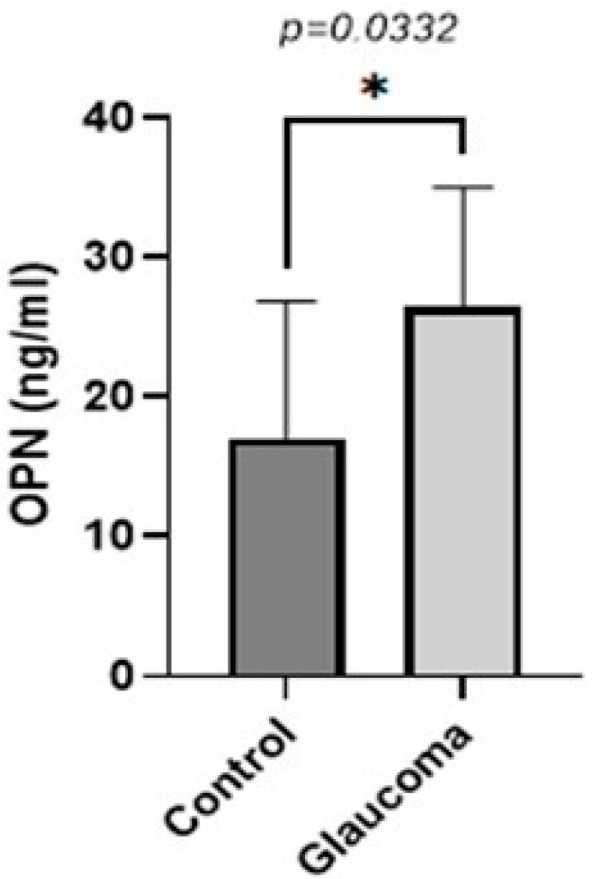
Graph depicting the mean ± SD expression of OPN in the aqueous humor of control (n = 16) and glaucoma (n = 16) patients with ELISA: OPN levels measured via an immunoenzymetric assay were significantly higher in the glaucoma patients with an average of 26.5 ng/mL ± 8.5, compared to the control group (*p* = 0.0332). Statistical analysis was performed using an unpaired *t*-test. Statistically significant difference: * (*p* < 0.05).

The levels of five biomarkers (OPN, MMP-9, TNFA, TGFB, IL-10) were quantified in all AH samples. OPN levels were higher in glaucoma groups compared to the control group (*p* < 0.05, unpaired *t*-test; Figure 1). The AH of POAG patients contained 1.55 ± 0.8-fold more OPN compared to that of control patients. In controls, it was 17.09 ± 10.11, whereas in the glaucoma AH, this was increased to 26.50 ± 8.50.

**Figure 2:**
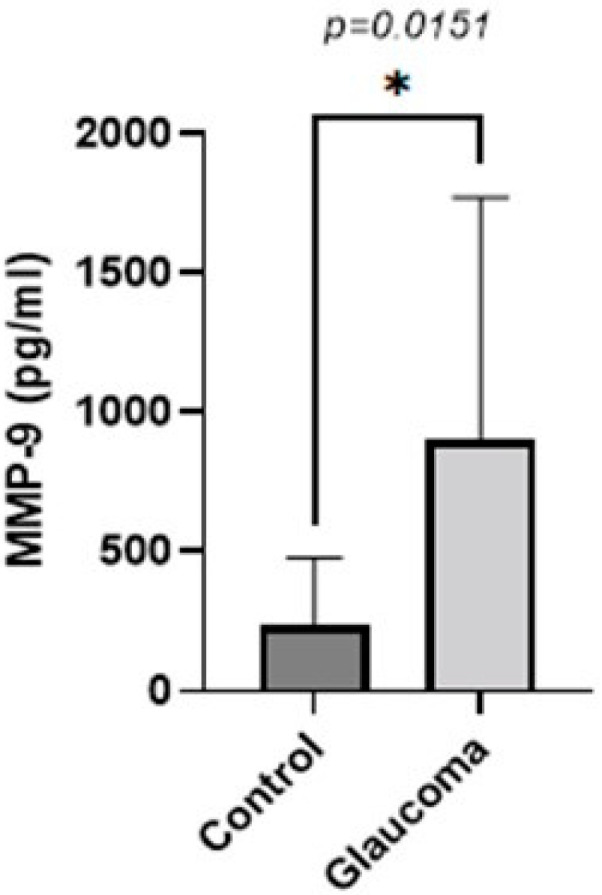
Graph depicting the mean ± SD expression of MMP-9 in the aqueous humor of control (n = 16) and glaucoma (n = 16) patients with ELISA: MMP-9 levels measured via an immunoenzymetric assay were significantly higher in the glaucoma patients with an average of 0.904 ± 0.86 ng/mL, compared to the control group (*p* = 0.0151). Statistical analysis was performed using an unpaired *t*-test. Statistically significant difference: * (*p* < 0.05).

Similarly, the concentration of MMP-9 in the AH was higher in the glaucoma group compared to the control group (*p* < 0.05, unpaired *t*-test; Figure 2). The AH of POAG patients contained 3.7 ± 3.6-fold more MMP-9 compared to that of control patients. In controls, it was 241.10 ± 235.08 g/mL, whereas in the glaucoma AH, this was increased to 904.5 ± 869.21.

**Figure 3:**
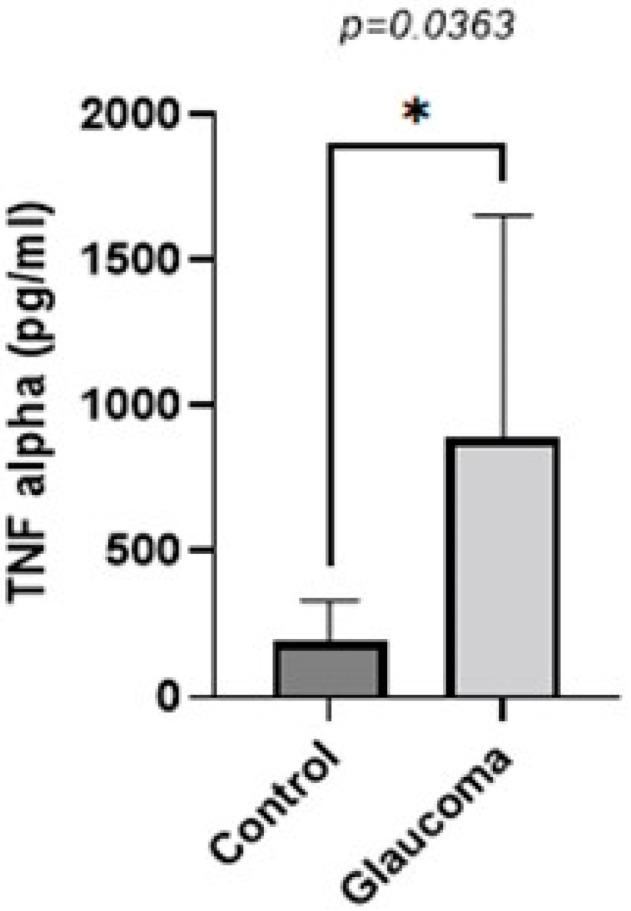
Graph depicting the mean ± SD expression of TNF-alpha in the aqueous humor of control (n = 16) and glaucoma (n = 8) patients with ELISA: TNF-alpha levels measured via an immunoenzymetric assay were significantly higher in the glaucoma patients with an average of 0.89 ± 0.36 ng/mL, compared to the control group (*p* = 0.0363). Statistical analysis was performed using an unpaired *t*-test. Statistically significant difference: * (*p* < 0.05).

TNF-alpha levels were higher in the glaucoma group compared to the control group (*p* < 0.05, unpaired *t*-test; Figure 3). The AH of POAG patients contained 4.55 ± 2.6-fold more TNF-alpha compared to that of control patients. In controls, it was 196.41 ± 135 pg/mL, whereas in the glaucoma AH, this was increased to 895.04 ± 362.09.

**Figure 4:**
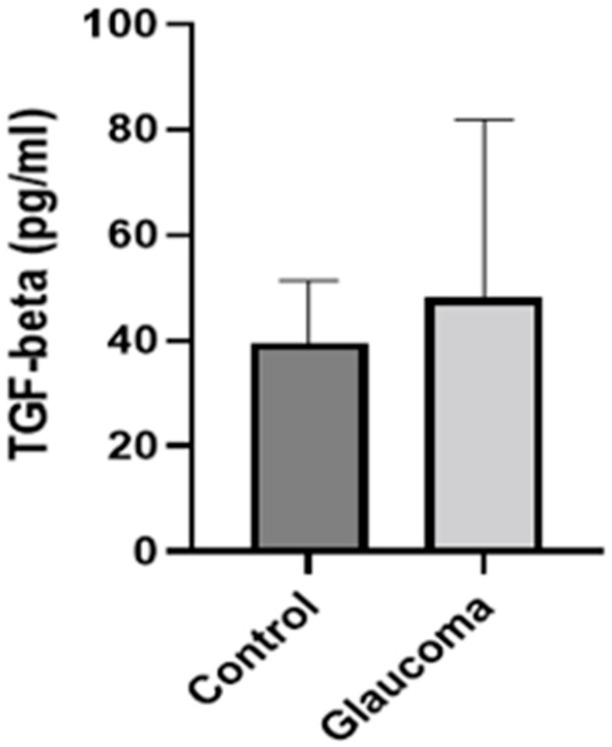
Graph depicting the mean ± SD expression of TGF-beta in the aqueous humor of control (n = 8) and glaucoma (n = 13) patients with ELISA: TGF-beta levels measured via an immunoenzymetric assay were not significantly higher in the glaucoma patients, compared to the control group.

**Figure 5:**
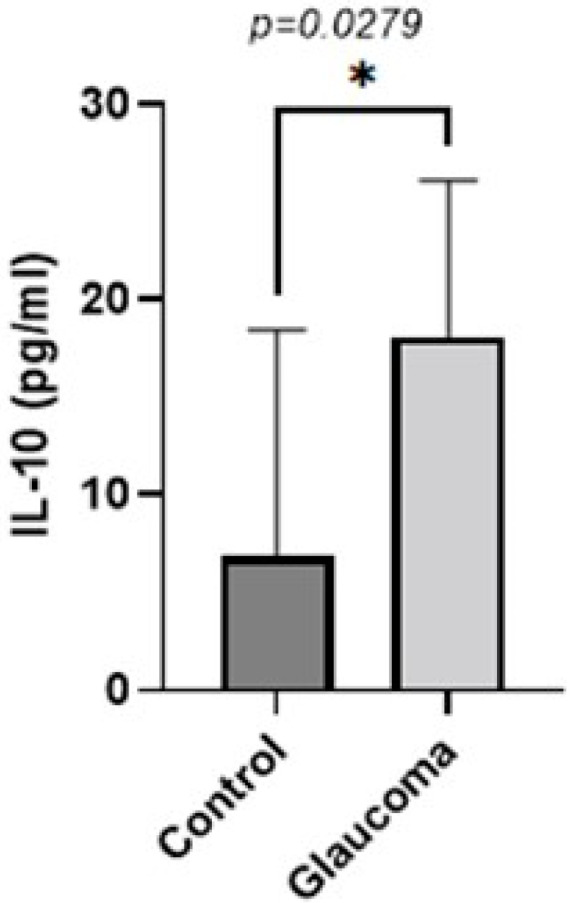
Graph depicting the mean ± SD expression of IL-10 in the aqueous humor of control (n = 8) and glaucoma (n = 8) patients with ELISA: IL-10 levels measured via an immunoenzymetric assay were significantly higher in the glaucoma patients with an average of 18.125 ± 8.21 pg/mL, compared to the control group (*p* = 0.0279). Statistical analysis was performed using an unpaired *t*-test. Statistically significant difference: * (*p* < 0.05).

There was a small difference in mean TGF-beta 1 expression between glaucoma and control groups. In control, it was 39.62 ± 11.6 pg/mL, whereas in the glaucoma AH, this was increased to 48.15 ± 33.73 (NS; Figure 4). IL-10 levels were higher in the glaucoma group compared to the control group (*p* < 0.05, unpaired *t*-test; Figure 5). The AH of POAG patients contained 4.55 ± 2.6-fold more IL-10 compared to that of control patients. In controls, it was 6.87 ± 11.57 pg/mL, whereas in the glaucoma AH, this was increased to 18.13 ± 8.21. The mean and SD for each sample with *p*-values of the selected biomarkers between the control and glaucoma groups are shown in Table 2.

TGF-beta concentration in the aqueous humor of control and glaucoma patients with ELISA: TGF-beta levels measured via an immunoenzymetric assay were not significantly higher in the primary open-angle glaucoma patients (POAG90) group compared to the control group. The group average was calculated according to the number of samples included in the TGF-beta ELISA assay, compared, and analyzed using an unpaired *t*-test.

For all biomarkers, there is an increase in the AH expression between the control and glaucoma groups.

### 3.3. Correlation Analysis of Biomarker Concentration and Postoperative IOP Values in Glaucoma Patients

**Figure 6:**
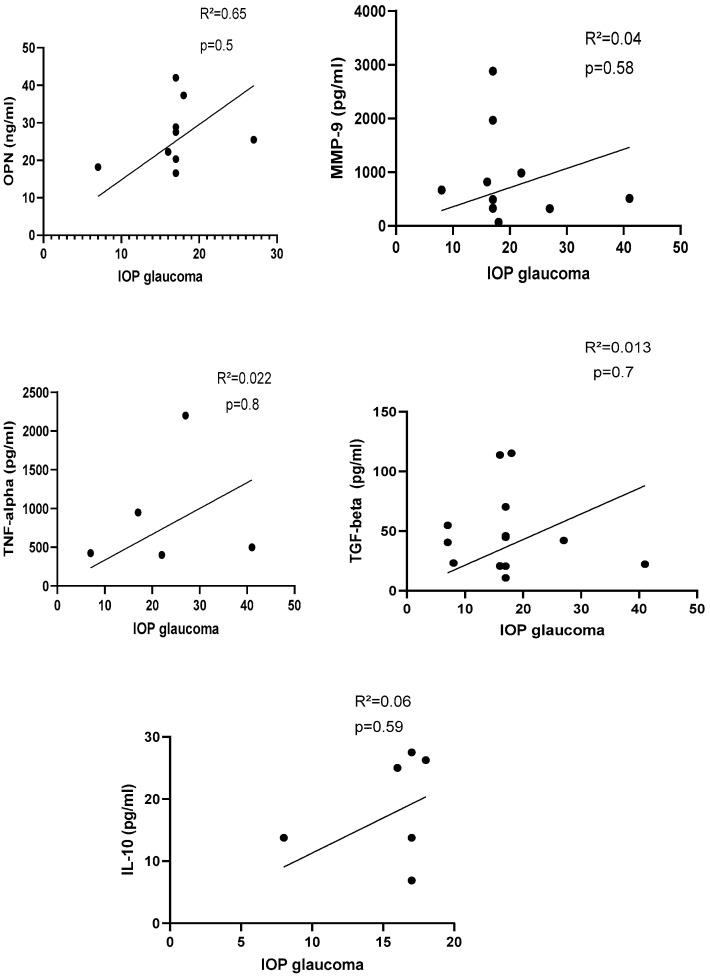
Correlation between protein biomarker and IOP in glaucoma group. IOP with OPN: *R*^2^ = 0.65, *p* = 0.5 (n = 9); IOP with MMP-9: *R*^2^ = 0.04, *p* = 0.58 (n = 10); IOP with TNF-alpha: *R*^2^ = 0.022, *p* = 0.8 (n = 5); IOP with TGF-beta = 0.013, *p* = 0.7 (n = 12); IOP with IL-10: *R*^2^ = 0.06, *p* = 0.59 (n = 6). IOP: intraocular pressure, OPN: osteopontin, TNF-α: tumor necrosis factor-alpha, TGF-beta: transforming growth factor-beta, IL-10: interleukin-10. *p*-Values were calculated with Pearson correlation, and a significant difference was defined when *p* < 0.05.

Preoperative and postoperative IOP values were recorded for patients in each group (Table 1), and a Pearson correlated analysis was performed to check the correlation between each biomarker with postoperative IOP (Figure 6). Patients with glaucoma had higher IOP than patients selected as controls.

There was no correlation between selected protein biomarkers and IOP in glaucoma patients (n = 16; Figure 6).

### 3.4. Comparison of Osteopontin Concentration in AH between Males and Females

Osteopontin concentration was compared by sex (Table 3).

Female glaucoma patients exhibited a 1.09 ± 0.9-fold lower OPN level compared to male glaucoma patients but the difference was not significant (22.34 ± 10.21 ng/mL versus 24.39 ± 9.41 ng/mL, *p* = 0.749). Females in the control group showed higher concentrations of OPN compared to the male control sample (25.69 ± 19.02 ng/mL versus 15.74 ± 12.13 ng/mL, *p* = 0.22). The mean age in the female control group was 54.5 ± 7.12 years and the mean age in the male control group was 55.37 ± 6.53 years with no significant difference between the two groups.

No difference was found (*p* = 0.51) when OPN values were compared between all males (23.24 ± 7.15 ng/mL) and all females (26.6 ± 14 ng/mL) (i.e., control and glaucoma groups were combined).

## 4. Discussion

Glaucoma progression depends on the glaucoma subtype. A high IOP is one of the clinical symptoms and used to characterize some glaucoma subtypes. IOP can vary with age and different co-morbidities, affecting the exact diagnosis for the glaucoma subtype [18]. There is a key unmet need for more objective criteria, such as biomarkers, that can help to stratify glaucoma by subtype and may assist with disease diagnoses. This study evaluated five analytes with potential as clinically relevant biological markers to aid glaucoma diagnoses. This study compared levels of the analytes in individuals with and without glaucoma, presenting for cataract surgery. The main findings of this study are the higher concentrations of the selected proteins (except TGF-beta) in the glaucoma group, and the lack of correlation between the concentrations of the selected biomarkers and postoperative IOP in the glaucoma group.

These results demonstrate increased expression of selected biomarkers related to oxidative stress and inflammation in patients with POAG compared to control subjects. A panel of five biomarkers, identified in the literature, was found to be upregulated in the AH samples of glaucoma patients compared to controls.

It has been demonstrated that the pathogenesis of glaucoma is associated with mitochondrial dysfunction, which is also related to aging and causes the accumulation in oxidative stress [19]. Recently, increasing evidence has shown that mitochondrial injury and oxidative stress are involved in the eye’s anterior chamber, mainly in TM cell damage in glaucoma [20]. Indeed, a mitochondrial complex I defect has been reported to be associated with the degradation of TM cells in POAG patients [21]. Our study found that the mean age in the glaucoma group was 67.37 ± 12.92 years and 54.94 ± 9.23 years for the control group above 40 years of age, consistent with previous studies [22]. A study has demonstrated that the female sex is associated with a higher risk of angle-closure glaucoma in Asian populations [23,24]; meanwhile, in POAG, sex was not found to be a risk factor [25]. In the control group, the risk of angle-closure glaucoma was equal in both females and males. In our study, no statistical difference between gender was found, due to the low number of study participants and exclusion criteria.

The glaucoma patients described in Table 1 showed a mean of baseline intraocular pressure (IOP) of 16.18 ± 2.9 mmHg and a mean of postoperative IOP of 17.5 ± 8.09. Glaucoma patients received IOP-lowering medications to prevent intraoperative complications in this study. Some patients were already taking glaucoma medication in the first place. This study showed no significant change between pre- and post-IOP values for the glaucoma group (*p* = 0.546). However, there is a significant change between pre- and post-IOP values in the control patients (*p* = 0.015), due to the cataract surgery.

Several research groups have analyzed inflammatory biomarkers related to oxidative stress in aqueous humor samples. For example, OPN is highly upregulated in acute and chronic inflammatory pathways and has been implicated in several physiological and pathophysiologic processes such as the inflammation process, cell viability, and wound healing [26]. Changes in OPN concentration in the aqueous humor have been detected in patients with POAG compared to controls. OPN has been demonstrated as the most abundant protein in the AH, with a high level of expression [27]. In our study, OPN was the most expressed protein in the AH with the highest concentration on a nanogram scale, whereas the rest of the biomarkers were on a picogram scale. OPN may be involved in upregulated harmful processes such as ischemia, inflammation, or increased IOP. Moreover, it is potentially upregulated in glaucoma patients to protect RGCs, as seen in a mouse model [16]. However, persistent recurrence of these harmful processes could generate a constant OPN overproduction.

Consequently, the local accumulation could convert this protective effect into a degenerative development. In this case, OPN is considered the potential prognostic marker for neurodegenerative diseases and their severity [14]. In harmony with these studies, our results show a statistically significant difference in OPN abundance in the aqueous humor of POAG patients compared to control patients and confirm the potential role of OPN.

MMPs have essential roles in the outflow pathways of the eye and extracellular matrix turnover [28] by removing the extracellular matrix components and contributing to normal extracellular matrix development processes [29]. The involvement of MMPs, especially MMP-9, in the pathogenesis of various glaucoma types has been studied and discussed [30]. Hosseini M. et al. demonstrated the relative increase in MMP-9 in patients with POAG and showed that the content of MMP-9 is significantly higher in glaucomatous eyes. These changes have been analyzed in the aqueous humor of patients with POAG [31,32]. According to histological investigations, different studies have proved the critical role of connective tissues in glaucoma’s starting point and development within the mechanical concept of POAG disease [33].

In POAG, the formation of the extracellular matrix components is disturbed because the cornea and sclera’s elastic properties decrease [34]. The regulation of the extracellular matrix is needed to remedy this, and it is realized thanks to the proteolytic enzymes, particularly metalloproteinases, and specific inhibitors of their activity that are tissue inhibitors of metalloproteinases (TIMP). However, this regulation generates excessive MMP synthesis and causes pathological remodeling processes characterized by the imbalance between new fiber synthesis and old loss [35,36].

This study found a higher level of MMP-9 in the AH of POAG patients. An increase in the content of MMP-9 locally could indicate dysregulation in the cell remodeling processes, which contributes to the formation of autoimmune inflammation [35]. The relationship between an increase in intraocular pressure (IOP) and the development of RGC apoptosis in the context of MMP-9 has been previously evaluated. An association between increased MMP-9 levels, apoptosis, and increased IOP in glaucoma was found [37]. In our study, the increase in IOP in the glaucoma group was not significant (*p* = 0.456, n = 16). The absence of IOP changes could be explained by the fact that glaucoma patients were taking IOP-lowering glaucoma medication. In addition, in our study, IOP was not increased for the glaucoma group, but increased MMP-9 was found in the AH of POAG patients. This could be explained by the upregulation of glaucoma medication.

The cytokine TGF-beta 1 influences the activation of MMPs [38]. Conversely, it has been posited that MMP-9 contributes to TGF-beta activation by releasing it from the matrix [39]. Levels of TGF-beta 1 were reported as higher in the AH of POAG patients [18,40]. Our data also showed a non-significant increase in TGF-beta 1 in the AH of POAG patients, compared to controls (*p* = 0.397, n = 13). In other studies, AH TGF-beta 1 levels were significantly higher in patients with other forms of glaucoma (SOAG and XFG). TGF-beta 1 was proposed to stimulate ECM deposition, increasing the outflow resistance mechanism, which agrees with the known role of TGF-beta 1 in many fibrotic disorders [41].

Increases in TNF-alpha have been reported in the retina samples and optic nerve of glaucoma patients [42]. In our study, the concentration of this cytokine in the AH of patients with POAG is relatively higher than in control patients (*p* = 0.036). TNF-alpha levels are related to retinal ganglion cell apoptosis, and the higher level in the AH of glaucoma patients may be associated with increased outflow resistance [9,43]. TNF-alpha has been implicated as a critical modulator of the neuroinflammatory response in glaucomatous neurodegeneration, contributing to optic nerve damage [44]. Several in vitro studies report the elevation in TNF-alpha protein and gene expression in microglial cells. In a mouse model of glaucoma, a rapid upregulation in TNF-alpha was caused by microglial activation and retinal ganglion cell loss. This finding suggests that TNF-alpha-related cell death could be a potential mechanism contributing to glaucomatous damage.

ELISAs have previously been used to detect the presence of TNF-alpha in glaucoma and cataract patients. TNF-alpha was more frequently detected in glaucoma patients compared to cataract patients with no glaucoma. In addition, TNF-alpha levels were not associated with IOP or the severity of glaucoma. This indicates that TNF-alpha may not directly influence the IOP or severity in glaucoma. However, pathophysiological processes seen in glaucoma, such as acute ischemia, and cell damage can lead to injury and activation of glial cells. TNF-alpha is released by these activated glial cells, contributing to the neuroinflammatory response in glaucoma.

Only one patient included in the glaucoma group had a medical history of platelet disorders and received anticoagulant medication. Such medication to treat thrombosis and platelet disorders may inhibit inflammation-induced thrombosis [45]. High levels of TNF-alpha seem to indicate an increased risk of thrombotic events [46]. However, antiplatelet medication reducing inflammation-induced thrombosis through inhibition of HMGB1/NFκB/NLRP3 signaling may attenuate TNF-alpha levels [46]. Antiplatelet medication could not be directly related to glaucoma and may not affect the eye and the AH. Future studies should be realized to validate this medication in glaucoma.

The pro-inflammatory cytokine IL-10 has been described as more highly expressed in the aqueous humor of patients with POAG relative to healthy controls [8]. Cytokines of the IL-10 family are essential for maintaining epithelial layer integrity and facilitating tissue healing [47]. IL-10 is involved in fibrosis and described as a tissue remodeling-related inflammatory mediator, highlighting its potential pathophysiological mechanism in glaucoma. In the current data, the level of IL-10 in the aqueous humor was in the same range (mean of 18.13 ± 8.21 pg/mL in the glaucoma group and 6.87 ± 11.57 in the control group) compared to the studies reported by Csősz et al. and Burgos-Blasco B. et al. (mean of 10.71 ± 3.16) [8,48]. In this study, a higher level of IL-10 was found in POAG patients compared to the control group.

The concentration of inflammatory cytokines is challenging to compare between research laboratories and studies due to different factors such as variability in patient disease progression, age differences, alternative profiles of pharmacological treatment, and different analytical techniques of cytokine detection [49,50,51]. For instance, in our study, biomarker concentration was determined with the ELISA technique, whereas other studies used multiplex techniques. Nonetheless, recent studies, including the present one, have demonstrated an inflammatory response in the aqueous humor of POAG patients compared to the control group [51]. Ghasem Fakhraie et al. studied the association between three promoter polymorphisms of the IL-10 gene with susceptibility to pseudoexfoliative glaucoma (PEXG), POAG, and pseudoexfoliation syndrome (PEX). In a Chinese population, IL-10 polymorphisms were predictive factors for POAG pathogenesis [52]. It suggests that POAG could be detected in early-stage disease if at-risk individuals with specific IL-10 genotypes are identified. Consequently, an increased understanding of the role of IL-10 in the POAG eye disorder may open the door to future treatments [53].

In our study, patients with infection or ocular surgery other than cataract surgery within the last 6 months were excluded. Infection and surgery may play an important role in controlling the inflammatory biomarker release in both glaucoma and control groups. This study was designed to minimize the impact of inflammation and immune responses from other indications on the study results. Inflammation can be stimulated by the immune response, leading to side effects such as increased IOP and altered visual field measurements. These side effects can impact the accuracy and reliability of study measurements, making it difficult to interpret the results. Consequently, we created a more homogeneous study population with reduced variability, by excluding these patients, because infections and ocular surgeries can trigger an immune response and inflammation in the eye.

The immune response can potentially affect the baseline measurements of biomarkers related to inflammation and study outcomes. It has been described, according to the different recent literature, that the immune system plays a role in glaucoma pathogenesis by impacting the adaptive immune system, such as the change in antibody levels, T cells and lymphocytes, retinal microglial cells, and macrophages. Moreover, research has shown that immune responses in the retina, such as the activation of microglia and the production of cytokines, can lead to retinal damage [54].

The exact mechanism of glaucoma pathogenesis remains unknown. POAG is associated with increased IOP. The molecular mechanism in retinal ganglion neuropathy in POAG remains poorly understood. However, recent studies have demonstrated that biomarkers related to oxidative stress mechanisms and inflammation could be associated with IOP elevation. A potential biomarker, linoleate-derived oxidation products, was successfully quantified in AH samples from patients with glaucoma and cataracts [55]. Freedman and Iserovich [56] demonstrated that intraocular cytokine concentrations are increased, and the IOP is elevated, in the AH of glaucoma patients. Consequently, this increase in the concentration of inflammatory and oxidative stress markers may influence the AH outflow dynamic, leading to IOP elevation. In this context, IOP may cause the production of inflammatory cytokines in eyes with glaucoma [57].

Takai et al. investigated TGF-beta 1 in the AH of POAG patients and found a significant correlation with the elevation in IOP, suggesting the critical role of TGF-beta in IOP elevations [58]. Levels of matricellular proteins were also significantly elevated and positively correlated to IOP in acute primary angle-closure patients [59].

Tong et al. [60] analyzed cytokines as biomarkers to evaluate the severity of glaucoma and did not find a significant correlation of cytokine concentration in the AH with IOP values. This study found no significant correlation for any of the studied biomarkers within the study population. This result is probably due to the small population too, as found in this study, describing the same lack of correlation.

IOP monitoring and management play a crucial role in the diagnosis and treatment of glaucoma. While IOP remains a primary focus in glaucoma management, there is growing recognition of the involvement of inflammation in the pathogenesis and progression of the disease. Inflammatory biomarkers may still provide valuable insights into glaucoma and contribute to more effective monitoring and management strategies, especially when it comes to correlating IOP measurements and biomarker results. The correlation needs to be made in a larger population, where patients are tightly controlled for the disease stage and medication.

Elevated levels of the selected inflammatory biomarkers have been found in the aqueous humor of POAG patients, in both this study and others, indicating the presence of inflammation in the eye. Monitoring biomarker levels may help to identify individuals at a higher risk of developing glaucoma or early-stage disease progression, and eventually before the increase in IOP. However, the use of biomarkers cannot be realized from AH samples, as one would not be able to obtain them from all patients.

In addition, the higher concentration can aid in assessing the inflammatory status and guiding therapeutic interventions.

In recent studies, oxidative stress-related biomarkers have been correlated with increasing IOP in POAG patients. Oxidative stress modifies the structure of the extracellular matrix of the trabecular meshwork. Consequently, the aqueous humor outflow is disrupted, leading to the increase in IOP [61].

Biomarkers could be used in the future, in combination with other clinical assessments and imaging techniques, to improve the accuracy of diagnoses, monitor disease progression, and guide treatment decisions in glaucoma management.

In this study, the medical history of the enrolled patients captured eye diseases such as myopia and hypermetropy. Several patients receive medication that induce adverse effects like dry eye disease (DED), an ocular surface disease. DED and glaucoma are two distinct eye conditions, both involving inflammation and oxidative stress, that can coexist in individuals. The inflammatory processes associated with DED could potentially impact our selected biomarkers [62]. Inflammation plays a central role in DED, involving various inflammatory biomarkers such as cytokine IL-10, TNF alpha, and matrix metalloproteinases (MMPs), including MMP-9 [63]. These could cause synergistic inflammation when both dry eye disease and glaucoma involve inflammatory biomarkers.

For MMP-9, the inflammatory response in DED can affect the expression and activity of MMPs, which may have downstream effects on the extracellular matrix remodeling in the trabecular meshwork in glaucoma. Measurement of the ocular surface MMP-9 level provides a useful marker for inflammation in DED and POAG [64] as DED has a significant impact on the concentration of MMP-9.

OPN is a matricellular protein with several biological functions [65,66]. OPN can be considered a pro-inflammatory cytokine with immune response functions [67,68] and OPN exhibits protective roles in wound healing [69] and biomineralization [70] in chronic inflammatory diseases.

The impact of gender on OPN action remains unknown [71] and the potential influence of OPN genetic variants on expression levels is not well described [72]. Previously, estrogen has been shown to inhibit kidney stone formation by increasing kidney OPN expression, while testosterone promoted kidney stone formation by suppressing kidney OPN expression [73]. Given that OPN expression is impacted by sex hormones, there is a possibility that OPN could vary between males and females.

This study did not find a significant difference between males and females in the glaucoma group and in the control group of all subjects. The study population’s small size and the population’s differences (ages, medication, medical history) may explain this lack of difference. However, the impact of OPN on diseases involving an inflammatory context should be further studied between males and females.

Further interdisciplinary studies are encouraged to better understand the sex differences seen in glaucoma and better target at-risk populations.

Although exclusion criteria for subjects in this research excluded systematic diseases related to inflammation due to infection and ocular surgery, the aging process could be a risk factor for increased systemic oxidative stress [74]. Additionally, in this study, the small sample size can cause a discrepancy in the results of markers compared with other published studies.

A combination of biomarkers, used diagnostically, may provide valuable information about the inflammatory processes involved in glaucoma and clinically aid in monitoring the effectiveness of therapeutic interventions. Such biomarkers provide valuable information for understanding the inflammatory processes occurring in glaucoma and tailoring treatment approaches accordingly. Developing such an effective diagnostic kit can significantly improve the early detection and management of glaucoma.

Other limitations in this study have been detected. First, baseline IOP data are needed. Most glaucoma patients had already been administered glaucoma eyedrops before the surgery. The preoperative and postoperative IOP was measured. In glaucoma, the mechanisms underlying the changes in the AH must be further studied through in vitro and in vivo studies in addition to clinical studies.

## 5. Conclusions

Increased expression of selected oxidative stress and inflammatory biomarkers was found in the AH of POAG patients compared to control patients presenting for cataract surgery. The higher level of the selected biomarkers in POAG patients likely reflects the glaucomatous disease state caused by local oxidative stress and damage with the blockage of AH outflow. This study needs to be repeated in larger populations. It is likely that any effective molecular diagnostic kit for glaucoma will involve a combination of biomarkers, such as those evaluated in this study.

OPN aqueous humor concentration was the most expressed marker in AH samples and could be considered a potential biomarker of the diagnosis of glaucoma, combined with other pro-inflammatory molecules analyzed in this research. No differences were detected between gender according to the level of OPN in the aqueous humor. However, this preliminary observation requires validation in larger case–control datasets.

## Figures and Tables

**Table 1:** Demographic characteristics of the patients included in this study. Student’s *t*-test was performed to compare the difference in preoperative IOP and postoperative IOP (mmHg).

Variables	Control (n = 16) Mean ± SD	Glaucoma (n = 16) Mean ± SD
**Age (years)**	54.94 ± 9.23	67.37 ± 12.92
**Gender**		
Male [n (%)]	8 (50%)	6 (37.5%)
Female [n (%)]	8 (50%)	10 (62.5%)
**Clinical characteristics**		
Preoperative IOP (mmHg)	16.06 ± 2.26	16.18 ± 2.9
Postoperative IOP (mmHg)	20.94 ± 5.64 (*p* = 0.015)	17.5 ± 8.09 (*p* = 0.546)

**Table 2:** *p*-Values and normalized values of the selected biomarkers. The Student’s *t*-test was performed to compare the difference in biomarkers in glaucoma and no-glaucoma group. The significance level was set at *p* < 0.05.

Biomarker	Control Group	Number of Patients	Glaucoma Group	Number of Patients	*p*- Values
**OPN (ng/mL)**	17.09 ± 10.11	16	26.50 ± 8.50	16	0.033
**MMP-9 (pg/mL)**	241.10 ± 235.08	16	904.5 ± 869.21	16	0.015
**TNF-alpha (pg/mL)**	196.41 ± 135	8	895.04 ± 362.09	8	0.036
**TGF-beta (pg/mL)**	39.62 ± 11.6	13	48.15 ± 33.73	13	0.397
**Interleukin-10 (pg/mL)**	6.87 ± 11.57	8	18.13 ± 8.21	8	0.029

**Table 3:** Comparison of the level of osteopontin between females and males in the studied population.

Biomarker	Glaucoma, Female (n = 10)	Glaucoma, Male (n = 6)	*p*-Values
OPN (ng/mL)	22.34 ± 10.21	24.39 ± 9.41	0.74
**Biomarker**	**Control,** **Female (n = 8)**	**Control,** **Male (n = 8)**	***p*-Values**
OPN (ng/mL)	25.69 ± 19.02	15.74 ± 12.13	0.22
**Biomarker**	**Control + Glaucoma** **Female (n = 18)**	**Control + Glaucoma** **Male (n = 14)**	***p*-Values**
OPN (ng/mL)	26.60 ± 14.06	23.24 ± 7.15	0.51

## Data Availability

Data available in a publicly accessible under request. The data presented in this study are openly available under request.
